# Thermal Injury to the Subhepatic Appendix Following Percutaneous Ultrasound-Guided Radiofrequency Ablation for Hepatocellular Carcinoma: A Case Report

**DOI:** 10.3390/diagnostics13213322

**Published:** 2023-10-26

**Authors:** Eun Ju Yoon, Jin Woong Kim, Jun Hyung Hong, Sang Gook Song, Hyun Chul Kim, Young Hoe Hur, Hyung Joong Kim

**Affiliations:** 1 Department of Radiology, Chosun University Hospital and Chosun University College of Medicine, Gwangju 61453, Republic of Koreasgsong71@gmail.com (S.G.S.); khcikim@hanmail.net (H.C.K.); 2 Department of Hepato-Biliary-Pancreas Surgery, Chonnam National University Hwasun Hospital and Chonnam National University Medical School, Gwangju 61469, Republic of Korea; 3 Department of Biomedical Engineering, Kyung Hee University, Seoul 02447, Republic of Korea

**Keywords:** radiofrequency ablation (RFA), hepatocellular carcinoma (HCC), subhepatic appendix, fistula, anatomical variations

## Abstract

We present the first documented case of a fistula between the treated zone and the appendix after RFA in a patient with HCC. Contrast-enhanced CT and MRI revealed a subcapsular hepatic nodule with image findings of HCC located adjacent to the ascending colon and cecum. An ultrasound-guided core needle biopsy was subsequently performed to distinguish between hepatic metastasis and HCC. Post-RFA imaging identified a low-attenuating ablated area adjacent to an air-filled appendix. The patient later experienced complications, including increased liver enzymes and an abscess at the ablation site. Imaging revealed a fistulous tract between the RFA zone and the appendix. Over the following months, the patient underwent conservative treatment involving intravenous antibiotics and repeated percutaneous drainage, exhibiting eventual symptom relief and an absence of the fistulous tract upon subsequent imaging. This case highlights the rare complications that can arise during RFA due to peculiar anatomical variations, such as a subhepatic appendix, resulting from midgut malrotation and previous surgery. It is imperative for operators to be cognizant of potential anatomical variations when considering RFA treatment, ensuring comprehensive pre-procedural imaging and post-procedure monitoring. This case also emphasizes the potential viability of nonoperative management in complex scenarios in which surgical interventions pose significant risks.

**Figure 1:**
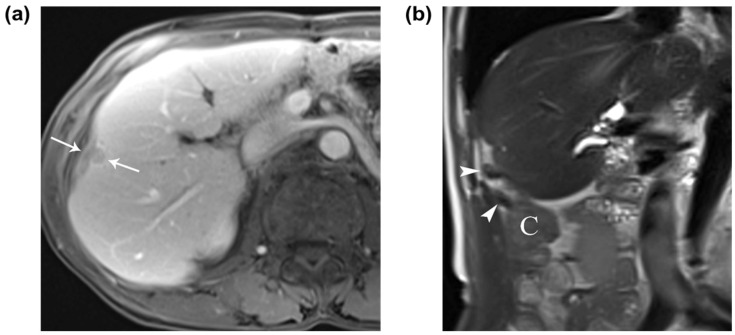
A 69-year-old man was admitted to our hospital for the evaluation and treatment of a hepatic mass. He had a history of pylorus-preserving pancreaticoduodenectomy and adjuvant chemotherapy for cholangiocarcinoma in the distal common bile duct 11 years previously and had developed liver cirrhosis due to alcohol abuse. Upon admission, laboratory findings were unremarkable. Contrast-enhanced computed tomography (CT) and gadoxetic-acid-enhanced magnetic resonance imaging (MRI) were performed to evaluate the hepatic mass. The MRI showed a 1.2 cm subcapsular hepatic nodule with typical findings of HCC in segment 5 of the liver, located adjacent to the cecum and ascending colon (Figure 1). Although the hepatic nodule has typical imaging findings of HCC, the possibility of hepatic metastasis cannot be ruled out. Thus, we decided to perform a percutaneous ultrasound (US)-guided core needle biopsy to distinguish between hepatic metastasis and HCC. RFA was selected as the treatment of choice after the nodule was confirmed to be HCC. RFA was performed under intravenous and local anesthesia. Before electrode placement, hydrodissection using 1 L of 5% dextrose solution was introduced under US guidance to minimize thermal injury to the adjacent colon. Tumor ablation was performed for 8 min after the placement of electrodes. A 1.2 cm subcapsular hepatocellular carcinoma in segment 5 of the liver in a 69-year-old man. (**a**) Contrast-enhanced axial T1-weighted MR image shows the washout of a 1.2 cm subcapsular hepatocellular carcinoma (arrows) on the portal venous phase. (**b**) A coronal T2-weighted MR image shows the perihepatic location of the appendix (arrowheads) and cecum (C) around the hepatocellular carcinoma.

**Figure 2:**
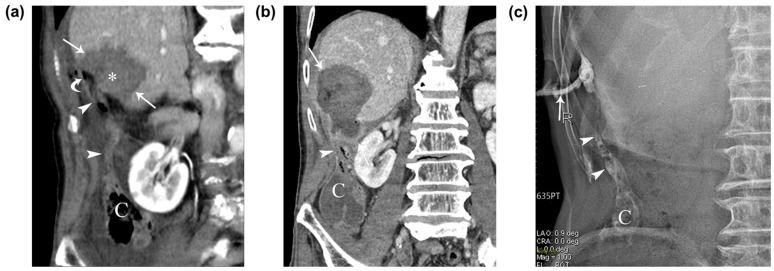
An immediate post-RFA contrast-enhanced CT scan demonstrated a low-attenuating ablated area that covered the tumor and the air-filled appendix, which extended superiorly from the cecum to the perihepatic space near the ablated zone of segment 5 of the liver, with a thickened appendiceal tip (Figure 2a). The patient was closely monitored and treated with intravenous antibiotics. On the fifth day after RFA, the patient’s laboratory results showed elevated aspartate aminotransferase and alanine aminotransferase levels, as well as an elevated high-sensitive C-reactive protein level. The patient presented with intermittent right upper abdominal pain and chills after the RFA, and a follow-up CT scan performed 7 days later revealed the formation of an abscess in the ablated zone and adjacent perihepatic space, although communication between the appendix and the ablated zone was not clear on the CT scan. A 10-French pigtail percutaneous drainage catheter was inserted into the hepatic abscess between the ablated zone and the perihepatic appendix (Figure 2b), and fluoroscopic imaging showed a fistulous tract between the ablated zone and appendix (Figure 2c). Abscess formation after percutaneous ultrasound-guided radiofrequency ablation for a 1.2 cm subcapsular hepatocellular carcinoma in segment 5 of the liver. (**a**) Immediate follow-up contrast-enhanced coronal CT image after ultrasound-guided radiofrequency ablation depicts a low-attenuating ablated area (arrows) which sufficiently covers the tumor (asterisk) and an air-filled perihepatic appendix (arrowheads) with a thickened tip from cecum (C). Note the small air density (curved arrow) between the ablated area and the appendiceal tip. (**b**) Follow-up contrast-enhanced coronal CT image taken 7 days after the RFA depicts abscess formation in the ablated zone and adjacent perihepatic space (arrow) and a thickened perihepatic appendix (arrowheads). (**c**) A 10-French pigtail percutaneous drainage catheter (arrow) was inserted into the sinus between the ablated area and the appendiceal tip. The fluoroscopic image depicts the contrast-filled appendix (arrowheads) with mottled air density and cecum (C). After 1 month, the patient’s clinical symptoms and laboratory findings subsided and improved, and a follow-up tubogram showed an improving abscess with a remaining fistula. Surgical treatment was initially considered, and the potential difficulties due to postoperative adhesion and potential postoperative complications were explained to the patient. However, the patient opted against this approach, leading us to decide on a conservative treatment. The patient was discharged following the removal of the percutaneous drainage catheter.

**Figure 3:**
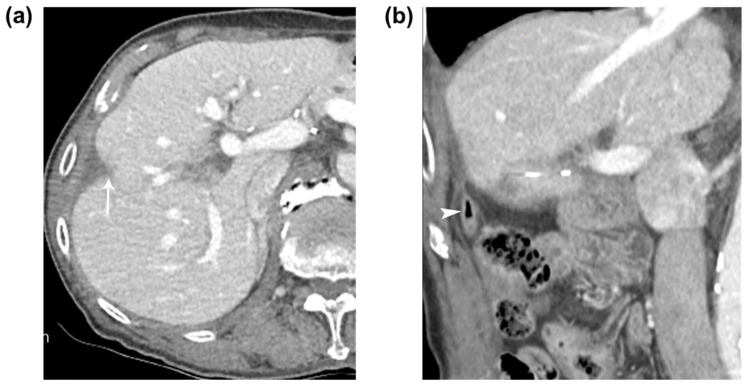
Eight months later, the patient was readmitted to the hospital for an evaluation of right upper abdominal pain. A CT scan revealed a remaining fistulous tract between the ablated zone and the appendiceal tip. The patient was treated with intravenous antibiotics and underwent repeated percutaneous drainage. After 1 month, the patient’s clinical parameters normalized, and a follow-up tubogram showed no communication between the appendix and the ablated zone. A follow-up CT scan showed the involuted RFA zone in segment 5 of the liver without a complete resolution of the abscess and recurrence (Figure 3). The patient underwent regular follow-up for 3 years without recurrence or any complication. Complete resolution of the abscess with a fistula between the previously ablated area and the appendix after 9 months. (**a**,**b**) Nine-month follow-up CT images showed the involuted RFA zone (arrow) in segment 5 of the liver and the intact appendix (arrowhead) with a complete resolution of the abscess or fistula. Percutaneous US-guided RFA is an effective treatment which is widely used for the treatment of HCC. The clinical outcome of RFA is influenced by major complications, one of which is thermal injury to non-targeted organs adjacent to the liver tumor. When performing RFA for tumors located in the inferior segment of the liver, such as the adjacent colon, thermal injury can occur [1,2,3,4,5,6]. However, in our case, there was the formation of an abscess or fistula between the appendiceal tip and the RFA zone due to collateral thermal injury that caused appendicitis as the subhepatic appendix was located adjacent to the RFA zone. The typical locations of the cecum and appendix are in the right lower abdomen, but due to anomalies and variations in midgut rotation, they can be located in the left part of the abdominal cavity, below the pylorus, or in the subhepatic space. When the elongation of the proximal colon does not extend sufficiently into the right lower abdomen in Phase III, subhepatic cecum and appendix can occur, which accounts for approximately 6% of all cases [7]. The positional variations of the appendix, such as its location and length, can lead to different clinical manifestations, inflammatory patterns, and complications, including abdominal pain [8,9]. In our case, the appendix and cecum were located in the right upper subhepatic space due to a variation in Phase III, with the appendix positioned in the caudocranial direction, traversing from the subhepatic space to the perihepatic space in the form of an inverse cecum that was relatively short and located posteriorly to the ascending colon. Additionally, a previous pylorus-preserving pancreaticoduodenectomy, which required the dissection of the right colon for duodenum and SMV exposure, likely caused adhesions between the cecum, appendix, and subhepatic space. Despite pre-treatment hydrodissection, collateral thermal injury occurred, leading to the formation of an abscess between the ablated zone and the appendiceal tip. The treatment for appendicitis typically involves appendectomy using laparoscopy as a primary option but in our case, surgical intervention was difficult due to adhesions caused by previous surgeries. Several articles suggest that nonoperative management could be considered for patients with perforated appendicitis [10,11]. Nonoperative management may be suitable for patients presenting with localized abdominal pain and a stable condition but not for those with diffuse abdominal pain or a deteriorating condition [12]. In our case, we started with nonoperative management using antibiotics and planned to perform frequent imaging follow-ups. After confirming the formation of an abscess via CT, we additionally performed percutaneous drainage guided by imaging. It is important to note that recurrence is most common within the first 6 months of follow-up in patients with perforated appendicitis who undergo nonoperative management, with a reported rate of recurrent appendicitis of 12.4% during the follow-up period [10]. Before performing RFA, imaging should be used to confirm the presence of heat-vulnerable organs around the tumor and to check for any anomalies or variations in the organs. Especially in cases like ours, in which the tumor is located in the inferior segment of the right hemiliver and there is a history of previous abdominal surgery, the position of not only the colon but also the cecum and appendix should be confirmed. To minimize thermal injury before the procedure, hydrodissection or the infusion of artificial ascites can be an effective strategy for successful RFA. However, despite aggressive pre-treatment, careful observation and diagnosis are important considering the possibility of fistula formation or abscess formation in the ablated zone and adjacent organs when thermal injury is suspected upon immediate follow-up CT. Nonoperative management should also be considered depending on the situation of the disease.

## Data Availability

The data presented in this study are available upon request from the corresponding author. The data are not publicly available for confidentiality reasons.
